# Development of a Soda-Lime Glass Feedstock for Injection Molding

**DOI:** 10.3390/ma19050854

**Published:** 2026-02-25

**Authors:** Martin Zürn, Steffen Antusch, Dorit Nötzel, Annika Schrage, Heinz Walter, Thomas Hanemann

**Affiliations:** 1 Institute for Applied Materials, Karlsruhe Institute of Technology, Hermann-von-Helmholtz-Platz 1, 76344 Eggenstein-Leopoldshafen, Germany; 2 Department of Microsystems Engineering, University of Freiburg, Georges-Köhler-Allee 102, 79110 Freiburg, Germany

**Keywords:** glass injection molding, soda-lime glass molding, PEG/PVB-based binder

## Abstract

**Highlights:**

**What are the main findings?**

- Development of new soda-lime glass containing feedstocks.
- Feasibility of injection moldability demonstrated.
- Dense glass parts after debinding and sintering obtained.

**What is the implications of the main findings?**

- Dense sintered soda-lime glass parts of different sizes can be realized by injection molding and thermal postprocessing.

**Abstract:**

Injection molding has been used for many years in the fabrication of thermoplastic parts with different complexities. With metal and ceramic injection molding, it is possible to realize at the end of the related process chain sintered metal and ceramic parts. Parts made from glass are rather seldom realized applying powder technology methods. This work describes the production of devices made from a commercial soda-lime glass applying the process chain of powder injection molding, covering the individual process steps like compounding, shaping, debinding, and sintering. In the first step, a binder consisting of polyethylene glycol (PEG) with different average molecular masses (4000, 8000, and 20,000 g/mol), polyvinyl butyral (PVB), and stearic acid (SA) were used for compounding new feedstocks with a solid load of 55 Vol% and 60 Vol%. As filler, a soda-lime glass with an average particle size of 6.1 µm, an almost symmetrical particle size distribution, a specific surface area of 0.78 m^2^/g, and a spherical morphology was applied. The measured equilibrium torque during compounding was low, with values between 2.5 and 5.5 Nm depending on the solid load and average molecular mass of the investigated PEG. All feedstock possessed a pseudoplastic flow behavior in the shear rate range between 10 and 3500 1/s. Small disk-shaped parts, as well as large cuboids and plates, were injection molded to a good quality. These green bodies were pre-debinded in water to remove the PEG, subsequently followed by thermal debinding to eliminate the remaining organic moieties. The concluding sintering in the temperature range between 660 and 680 °C delivered glass parts with huge density values close to 100% of the theoretical value, as measured by the Archimedes method. The principal feasibility of glass injection molding with a suitable feedstock system could be demonstrated successfully.

## 1. Introduction

From an applicational point of view, polymers, metal, and ceramics are the most relevant material classes worldwide. In addition, silicon and other semiconductors are essential for all microelectronic and photovoltaic devices. Beyond their usage in architecture, e.g., as facades, inorganic glasses are used in highly transmissive optical fibers, chemical reaction technology, health technology, or energy production such as in thermal solar power plants. The relative simple chemical composition based on silicon dioxide as a main component and different amounts of oxides, like boron, sodium, potassium, aluminum, calcium, magnesium, and many more, allows for properties to be tailored targeting different technical applications [1,2]. Typically, glass is shaped by the float or blow processes, but complex shapes cannot be realized in this way. In general, glass must be shaped in the molten state at elevated temperatures, which causes pronounced energy consumption accompanied with significant CO_2_ release [3,4]. Powder technology, as established for metals and ceramics, may deliver a suitable alternative for cost reduction by avoiding the usage of a large glass kiln and tank melting [5]. Powder injection molding (PIM) with the two main sub-processes metal and ceramic injection molding (MIM and CIM respectively) has allowed for the fabrication of metal and ceramic parts in excellent quality for many years [6,7,8,9]. PIM represents a process chain covering the individual steps of feedstock formation (compounding); shaping, like injection molding (IM); and thermal postprocessing, like debinding and sintering, to finalize the dense ceramic or metal part.

Recently, our group’s first investigations focused on the development of glass injection molding (GIM) applying a borosilicate glass as filler and an environmentally friendly, partially water-soluble binder consisting of polyethylene glycol (PEG), polymethylmethacrylate (PMMA), and stearic acid (SA) [10]. Small sintered glass parts with densities up to 100% th. could be obtained after a comprehensive evaluation of the PEG/PMMA/SA binder systems. The process development suffered from an irregular morphology of the investigated borosilicate glass particles, which affected feedstock preparation and characterization [10]. In this work, an extensive overview of the state-of-the-art GIM has also been given [10]. Consequently, only publications applying material combinations like this work are discussed below. Mader et al. [11] described a composite consisting of pure fused silica glass with PEG and PVB. To achieve good feedstock homogenization, all components were mixed in a solvent, which must be removed prior its usage in IM [11]. After vacuum sintering at 1300 °C, dense and transparent small fused silica parts could be obtained [11]. A feedstock containing recycled soda-lime glass, low-density polyethylene (LDPE), paraffin wax (PW), and stearic acid (SA) as a surfactant was investigated by Hidalgo et al. [12]. When using a feedstock with 65 Vol% glass filler, they achieved a sample density around 90% th. after sintering [12]. In more general work, the adaption of CIM to GIM has been described in [13]. The basics of the PIM process can be found in [14] and was revisited in 2025 [15].

Established binder systems for MIM and CIM consist of a low molecular mass polymer, like PEG or wax; a high molecular mass polymer, like LDPE or HDPE; PMMA and PVB; and a surfactant, like SA, as well as other related fatty acids [16,17,18,19,20]. In most cases, the low molecular mass polymer is removed in a liquid pre-debinding step using appropriate solvents, like hexane for wax and water for PEG, and the high molecular mass polymer is thermally debinded prior to sintering. With respect to environmental reasons, the usage of hexane and other related organic solvents should be avoided; therefore, PEG/PMMA- or PEG/PVB-based binders, or the combination of both, are gaining more importance in PIM and the related material extrusion (MEX) of metal- or ceramic-containing feedstocks [10,21,22].

In continuation of previous work [10], new feedstock systems containing commercial soda-lime glass, PEG, and PVB as binders in combination with SA as a surfactant were investigated. Due to the low melting temperature around 58–60 °C and the very low PEG melt viscosity of PEG, the resulting feedstock’s melt viscosity was significantly reduced, simplifying the molding process, especially the mold filling. PVB can act as the backbone polymer, which delivers a certain mechanical stability necessary for demolding the molding tool after IM. SA functions as a surfactant to enable good wetting of the glass filler by the polymeric binder, as well as a release agent against the metal mold inserts to prohibit adhesion during demolding. In the following work, a precise description of the process chain development showing the principal feasibility of successful injection molding and the realization of dense soda-lime glass parts will be given.

## 2. Materials and Methods

### 2.1. Material Selection

**Figure 1:**
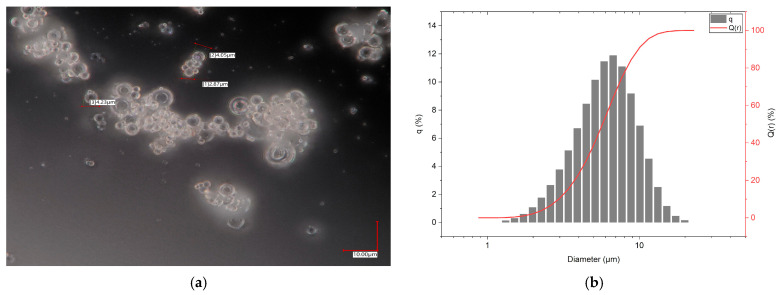
Swarcoforce soda-lime glass particles: (**a**) light microscopic image showing an almost spherical morphology; (**b**) measured particle size distribution (d_10_: 2.9 µm, d_50_: 6.1 µm, d_90_: 9.9 µm).

A commercial soda-lime glass of the container type, entitled Swarcoforce from Swarco Indusferica (Neufurth, Austria), was selected as a glass filler [23]. The almost spherical morphology is depicted in Figure 1a. The measured particle size distribution (volume-based, Figure 1b) shows particle sizes mostly between 1 and 20 µm with a d_50_—value around 6.1 µm (LA-950, Retsch Technology, Haan, Germany). The measurement of the specific surface area according to the BET method was 0.78 m^2^/g (Gemini VII, Micromeritics Instrument Corporation, Norcross, GA, USA). The type of Swarcoforce glass filler used was renamed and has now been made available under the name Swarcoforce 1-20 (Swarco Indusferica, Neufurth, Austria), possessing almost identical specification and composition. The composition of the Swarcoforce glass was measured by X-ray fluorescence analysis (S4 Pioneer, Bruker AXS, Karlsruhe, Germany). Origin Pro 2023 was used for the evaluation of the measured data.

A different series of new glass feedstocks were developed containing mixtures of the water-soluble PEG, PVB, and SA. The amount of SA was calculated according to the specific surface area (mg/m^2^) of the glass filler; a covering monolayer can be assumed for an SA amount around 2–3 mg/m^2^. Increasing SA amounts were compensated by an equivalent reduction in PVB. While the same PVB type (B30H, Kuraray, Hattersheim am Main, Germany) was used, different PEGs were applied to prove the influence of average molecular mass on the feedstock properties and processing conditions (Table 1). PEG/PVB binder systems were successfully applied in PIM and MEX [11,21,22,24].

### 2.2. Compounding and Rheological Characterization

The compounding of all feedstocks has been realized by applying a torque recording compounder (W50 EHT, Brabender, Duisburg, Germany), allowing an in-line torque recording during mixing and visualizing the compounding progress at a given temperature with the proceeding time. For the PEG/PVB binder system, a mixing temperature of 125 °C was set. All compounds were mixed for at least 1 h with a mixing speed of 30 rpm as used in previous work, e.g., in [10,21,22], inside a mixing chamber with a volume of 50 cm^3^. After compounding, the feedstocks were characterized with a high-pressure capillary rheometer (Rheograph 25, Göttfert GmbH, Buchen, Germany, capillary length 30 mm, capillary diameter 1 mm, L/D = 30) at 140 °C. The shear rate range was varied between 10 and 3500 s^−1^. The measured data were corrected by the Weißenberg–Rabinowitch method, as recommended by the rheometer vendor [25,26].

### 2.3. Glass Injection Molding

Glass injection molding was used for the fabrication of parts with different geometric sizes and shapes. Small round test structures with a diameter of 10 mm and a thickness of 2 mm were injection molded using a micro injection molding machine (Microsystem 50, Battenfeld, Kottingbrunn, Austria). The mold inserts were made from tool steel. The standard molding temperature was set to 140 °C as derived from the viscosity measurements. Larger parts with geometric sizes varying from 41 × 41 × 15 mm^3^ to 66 × 26 × 4 mm^3^ were realized on an Arburg 420 C injection molding machine (Arburg GmbH + Co. KG, Loßburg, Germany). The materials used for the mold inserts were hardened steel 1.2343 (X37CrMoV5-1) and brass. After feedstock injection, a dwell pressure was applied for approximately 10 s to compensate for feedstock shrinkage during cooldown. For defect-free demolding, a molding tool temperature around 20–30 °C was necessary because of the low melting temperature of the PEGs used.

### 2.4. Debinding and Sintering

The green bodies were debinded in a two-step process: liquid pre-debinding under ambient conditions for 16 h in water [24], followed by thermal debinding at very low increasing temperature rates in a chamber oven HT/28 (Carbolite Gero, Neuhausen, Germany) with a maximum temperature of 500 °C. After debinding, the final sintering was then applied using a Carbolite Gero RHF 1700 furnace (Neuhausen, Germany) with maximum temperatures between 660 °C and 680 °C. Both thermal treatments were performed under air atmosphere.

### 2.5. Sintered Glass Part Characterization

All sintered Swarcoforce glass parts were characterized with respect to the achieved sinter density, which was measured according to the Archimedes principle with a YDK01 balance Sartorius (Sartorius, Göttingen, Germany) using Equation (1) where ρ_solid_: density of the solid specimen; ρ_liquid_: density of water; m_air_: dry specimen mass; and m_liquid_: specimen mass in water. The Archimedes method used to measure density can be quite sensitive to systematic errors originating from different sources, like bubble adhesion or temperature fluctuations causing density changes; consequently, deviations attributed to the method can be up to 2%. Nondestructive examination using CT scans of selected samples (Phoenix v tome xs, General Electric, Frankfurt, Germany) can enable the visualization of inner defects like voids or cracks in the whole sample volume. The accessible spatial resolution was 15 µm (measuring time: 100 ms; voltage: 10 kV, current: 120 µA). Optical transmission measurements were carried out using a UV/Vis spectrometer (SPECORD S 600; Analytikjena, Jena, Germany).


(1) $$\rho_{solid}=\rho_{liquid}\frac{m_{air}}{m_{air}-m_{liquid}}$$

## 3. Results and Discussion

### 3.1. Feedstock Compounding

**Figure 2:**
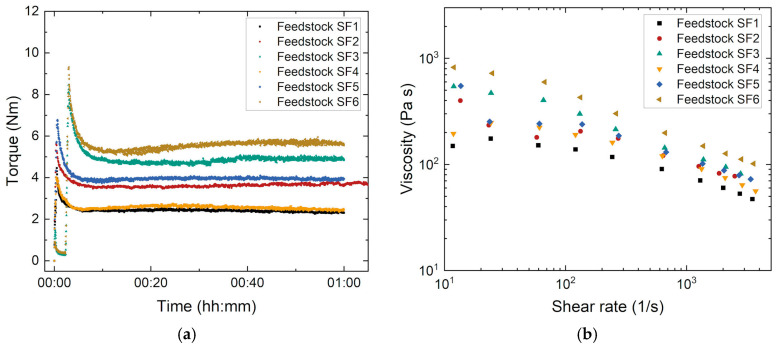
Characterization of feedstocks SF1–SF6: (**a**) compounding at 125 °C; (**b**) change in the melt viscosity with a shear rate at 140 °C.

Swarcoforce glass filler compounds with different solid loads (55 and 60 Vol%) and PEG with three different average molecular masses (4000, 8000, and 20,000 g/mol) were investigated. In all systems, the ratio of PEG and PVB was kept constant (1:1), as well as the SA amount of 35 mg/m^2^ (Table 2). Figure 2a shows the torque evolution with the proceeding compounding time measured at 125 °C. As described in detail in [10], the compounding process can be subdivided into three individual phases:

- Filling state: All thematerials were added into the mixing chamber in a defined order to retain the same experimental conditions. First, a fraction of the solid filler was filled in the chamber until the dough hooks were covered completely. Second, the surfactant was supported for an entire physisorption at the filler’s surface. Third, both PEG and PVB were added, and the remaining glass filler were placed inside. After that, the chamber was closed. Prior to full wetting, a maximum torque can be observed;
- Mixing state: Particle wetting by surfactant and organic binder was started. With increasing particle wetting of the glass filler by the surfactant, a pronounced torque drop can be observed. In an ideal case, all particles should be covered at least by one monolayer. Here, due to the very large SA content of 35 mg/m^2^, a multilayer coverage can be assumed;
- Stationary state: With the proceeding time, an equilibrium state between surfactant physisorption and desorption will be reached, delivering an almost constant torque value as evidence for complete surface coverage.

At a constant solid load, the equilibrium torque increased with the increasing average molecular mass of the PEGs. The change from PEG4000 to PEG8000 did not alter the torque significantly, but a pronounced torque increase can be detected with the change from PEG8000 to PEG20000, especially at the higher solid load. However, in contrast to feedstocks with alumina as a ceramic filler, the torque values were small, which can be attributed to the spherical particle shape of the glass (Figure 1a) and its small specific surface area. A solid load increase with a constant average mass of PEG caused a viscosity increase as well. Both were observed in previous investigations, e.g., described in [10].

### 3.2. Melt Viscosity

The knowledge about the temperature and shear rate dependent viscosity is relevant for shaping, like in PIM or Fused Filament Fabrication (FFF). While the equilibrium torque value can deliver information about feedstock homogeneity, the melt viscosity at certain shear rate ranges can give information about injection moldability. Figure 2b presents the evolution of the melt viscosity with shear rate at a constant temperature (140 °C). At low shear rates between 10 and approx. 70 1/s and with the usage of PEG4000 and PEG8000, the presence of the first Newtonian plateau representing the zero-shear viscosity η_0_ [25] can be adumbrated. At higher shear rates, all investigated feedstocks showed a shear-thinning (pseudoplastic) flow behavior, which is typical for highly filled feedstocks with ceramic or metal particles that are suitable for FFF and PIM [21,22]. In the cases of feedstocks SF3 and SF6 with the high molecular mass PEG (PEG20000), the first Newtonian plateau shifted to shear rates below the measured shear rate range starting at 10 1/s [25].

Due to the different process conditions in FFF and PIM, the effective shear rates were significantly different. While in PIM, shear rates up to several thousand per second, originating from the high injection speeds and injection pressures, can be measured, the filament in FFF can be conveyed by simple contact wheels without any remarkable pressure. As a result, typical shear rates in the nozzle were located between 50 and 100 1/s depending on the nozzle diameter, layer thickness, and filament feeding speed. Table 3 compares typical viscosity values for PIM and FFF resulting from the effective shear rates during processing. It can be clearly seen that due to the pronounced shear thinning, in PIM, significantly lower viscosity values can appear, which could simplify molding but hamper FFF. A closer view of the shear-rate-dependent viscosity ratio, which can be treated as a shear-thinning index [25], of the two different viscosity values (at small shear rate/at high shear rate) showed that the ratio was located around 2 when using PEG4000 or PEG8000. When using PEG20000, the ratio increased to a value around 3, which can be a hint for pronounced shear-thinning behavior; the higher solid load can also increase the viscosity ratio. In addition, preliminary printing trials showed the principal feasibility of FFF printing.

### 3.3. Process Chain for Small Test Specimen Fabrication

#### 3.3.1. Injection Molding

**Figure 3:**
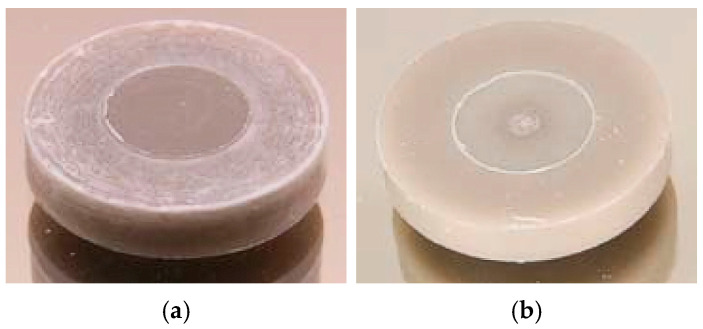
Injection-molded green bodies (disk with Ø 10 × 2 mm^2^) from different feedstock systems: (**a**) SF1; (**b**) SF3.

Injection molding of the mold inserts with different geometries was performed to evaluate the suitability of the new feedstocks for melt processing and shaping. In continuation of previous work dealing with feedstocks containing ceramic, glass, or metal developed for PIM and FFF [10,16,21,22], small test specimens were produced by PIM using the Microsystem 50 injection molding machine from Battenfeld. Figure 3a,b shows the slight brownish disk-shaped green bodies made from feedstocks SF1 and SF3, respectively.

In case of the low molecular mass of PEG4000 (Figure 3a) at the surface, a phase separation between the glass filler and the polymer can be assumed due to the presence of whitish textures. The use of PEG20000 with a high molecular mass yielded a more homogenous surface appearance (Figure 3b), and in the middle of the specimen the gate mark can be seen. In Table 4, the optimized injection molding parameters for the investigated feedstocks are listed. Due to the higher solid load in the cases of SF4–SF6, the cooling time was extended.

#### 3.3.2. Debinding and Sintering

**Figure 4:**
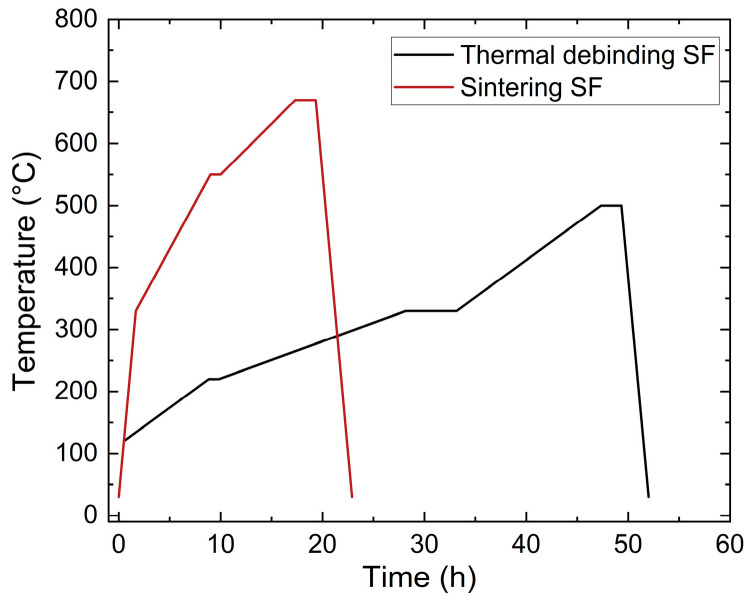
Temperature programs used for thermal debinding and sintering (small-sized samples).

According to previous investigations on PEG/PVB feedstocks which used zirconia as solid filler [24], a liquid pre-debinding was mandatory prior to thermal debinding. The specimens were placed in water under ambient conditions for 16 h and the PEGs as the low molecular weight binding component could be fully removed successfully. As the remaining large molecular component, the PVB was then removed thermally. This two-step process was a common practice in PIM and MEX according to the pore formation during the first debinding step, as it enabled the diffusion of gaseous polymer fragments from the bulk material without damaging the sample part in the second step [21,22]. During thermal debinding, the organic moieties decomposed into gaseous water and carbon dioxide, which was equivalent to a pronounced volume increase. Consequently, to avoid any damage on the samples, the heating rate during debinding should be very low, down to 1 °C/min, and dwell times should be considered for the pronounced decomposition of PEG and PVB. Hence, holding times at 220 °C (PEG, SA) and 330 °C (PVB) were selected to guarantee complete organic moiety decomposition [24]. The final heating up to 500 °C with a shorter holding time allowed for the removal of remaining binder traces. Figure 4 shows both the debinding and the sintering temperature programs. After debinding, sintering followed with a maximum sinter temperature between 660 °C and 680 °C.

Table 5 lists all the measured sinter densities of selected feedstocks by averaging all the investigated sinter temperatures between 660 and 680 °C. For a better comparison, only the small samples like the disks and sintered filament strands were considered. A notable difference in sinter density between the sintered filament strands and the printed parts could not be measured. The sintered samples from SF2, with a solid load of 55 Vol% and containing PEG8000 as a binder component, yielded a poor density value around 96% th. and a pronounced standard deviation; however, the switch to PEG20000 caused an increase of 99% th. (SF3).

SF6, with a solid load of 60 Vol% and PEG20000, showed a further increase in small density close to 100% th. In the latter cases, the calculated standard deviation dropped significantly. A systematic influence of the selected sinter temperature (660 °C, 670 °C, 680 °C) on the sintered parts density could not be detected.

**Figure 5:**
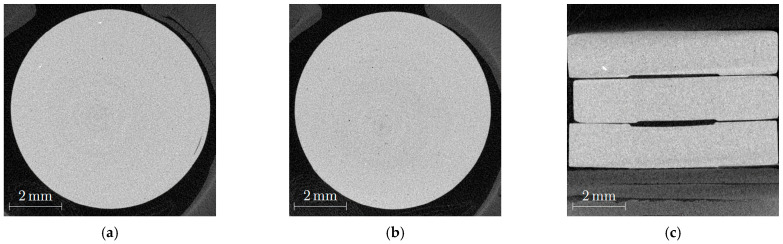
CT images of sintered specimens originating from feedstock SF6: (**a**) 670 °C for 2 h; (**b**) 680 °C for 2 h; (**c**) cross-section of three sintered parts (upper and middle parts sintered at 680 °C, lower part sintered at 670 °C).

The quality of the sintered parts was investigated using nondestructive CT scans (Figure 5). The influence of the sinter temperature on quality can be clearly detected in Figure 5a,b. Both images show a very small number of dark spots which can hint to the presence of voids that are equivalent with closed porosity. Closed porosity can be considered using the Archimedes method for density measurement, hence the found value of 100 ± 0.3% seems to be a little too high. In addition, the sample which was sintered at 670 °C exhibited a short crack on the right side. The comparison of samples sintered at both temperatures in Figure 5c shows a pronounced rounding of the edges in the case of the 680 °C sintered sample.

### 3.4. Process Chain for Large Test Specimen Fabrication

#### 3.4.1. Injection Molding

**Figure 6:**
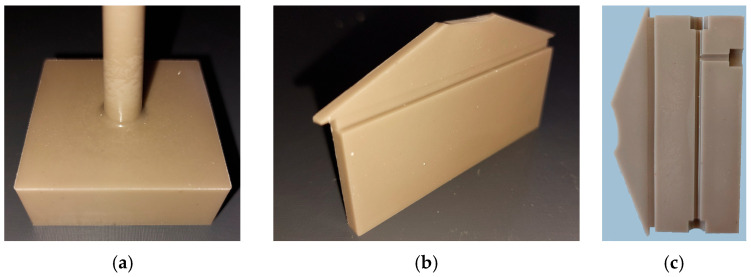
Images of larger injection-molded parts made from feedstock SF6: (**a**) cuboid with 41 × 41 × 15 mm^3^ size; (**b**) plate with 66 × 26 × 4 mm^3^ size, front side with film-like gate, (**c**) plate with 66 × 26 × 4 mm^3^ size, carrying a microfluidic structure on top.

With respect to different potential applications, larger parts with different geometries were realized by injection molding using SF6 as the optimal feedstock system with the highest investigated solid load of 60 Vol%. According to Figure 2b, the moderate melt viscosity at injection temperature (140 °C) should allow for the mold to be completely filled. Figure 6a shows a cuboid with the dimensions of 41 × 41 × 15 mm^3^. The green body possesses a smooth surface and sharp edges. Green parts with a more anisotropic shape can be seen in Figure 6b,c where the surface appearance is very plain and without visible cracks. All parts show a brownish color.

#### 3.4.2. Debinding and Sintering

**Figure 7:**
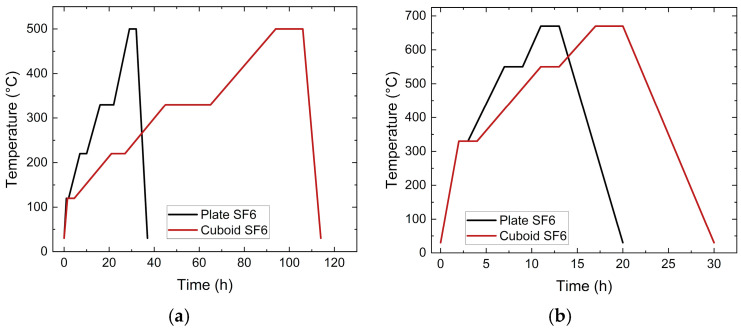
Thermal postprocessing of SF6’s large injection-molded parts: (**a**) thermal debinding; (**b**) sintering.

Due to the larger sizes accompanied with extended diffusion paths, the debinding and sintering procedures, especially the process times, must be adapted. In the case of the cuboid (Figure 6a), the sample was placed in water under ambient conditions for 12 days, and a 94.1% removal of PEG was measured. In the case of the thinner samples with a larger surface-to-volume ratio, as presented in Figure 6b,c, a liquid debinding duration time of 3 days was necessary. In both cases, a huge removal of PEG around 95% and 98% could be achieved. The subsequent thermal debinding program is shown in Figure 7a; in the particular case of the cuboid, the debinding time was significantly extended compared to the small test specimens. The final sintering step is displayed in Figure 7b, where the cuboid structure again needed an extended processing time. Finally, of the theoretical density with regard to the inaccuracy of method-related measurements, sinter densities around 97% (cuboid) and close to 100% (plates) could be achieved.

**Figure 8:**
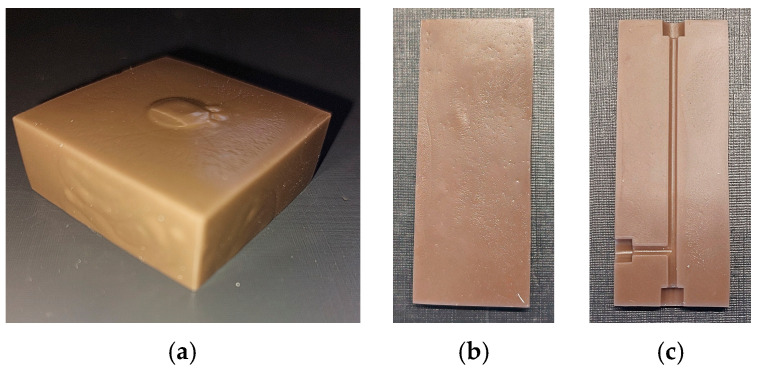
Images of larger sintered parts: (**a**) cuboid (35 × 35 × 12.5 mm^3^); (**b**) unstructured plate (55 × 22 × 3.5 mm^3^); (**c**) structured plate (55 × 22 × 3.5 mm^3^).

Figure 8 depicts the sintered parts; the brownish color of the green bodies was retained. While the gate-facing surface is quite course and the side walls of the cuboid show some swelling (Figure 8a), the quality of the unstructured plate has been improved, with the exception of some surface defects originating from the brass mold insert (Figure 8b). The best surface quality could be achieved with a new brass mold insert carrying microfluidic channels (Figure 8c).

**Figure 9:**
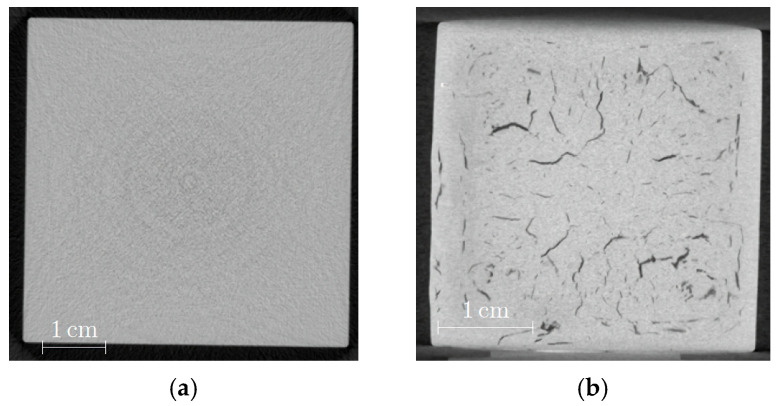
CT images of cuboid with a cross section in the middle: (**a**) green body; (**b**) sintered state.

**Figure 10:**
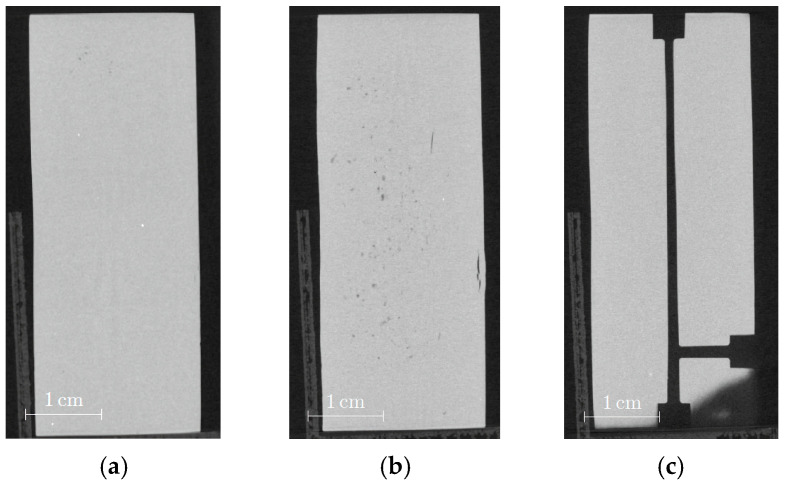
CT images of sintered plates: (**a**) unstructured plate close to the surface; (**b**) unstructured plate with a cross section in the middle; (**c**) structured plate close to the surface.

CT images can help to analyze the inner structure with respect to defects, like voids or cracks, which could reduce mechanical stability. Figure 9a presents a cross section of the green body, and no remarkable inner flaws can be observed. In contrast, Figure 9b shows the same sample after debinding and sintering with a huge number of extended cracks. It is known from experience that the debinding process in particular can cause pronounced defect generation due to polymer swelling during liquid pre-debinding, as well as due to non-optimized parameters during thermal debinding. These defects cannot be healed during sintering. Another reasonable explanation could be the release of inner stress during debinding. The CT images of the plate-shaped samples do not exhibit any defects close to the surface (Figure 10a,c); however, some voids and cracks can only be observed in the middle of these samples (Figure 10b).

### 3.5. Optical Transmittance

**Figure 11:**
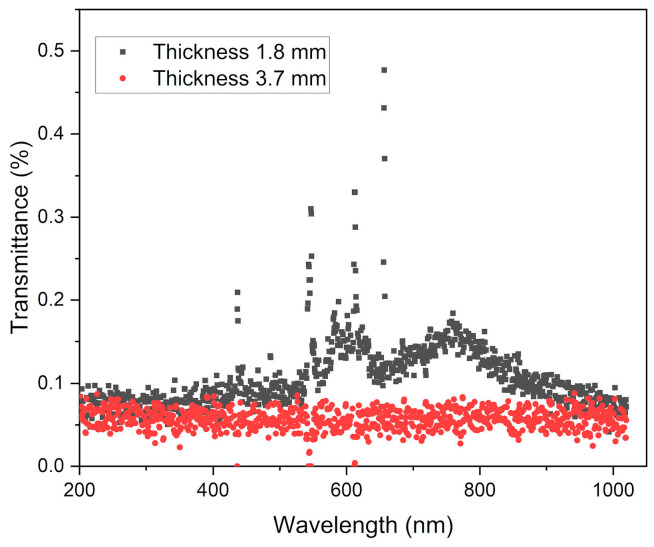
Optical transmittance spectra of two different (disk-shaped sample with 1.8 mm thickness and plate-shaped sample with 3.7 mm thickness) sintered Swarcoforce samples.

The measurement of the optical transmittance in the visible range produced very low values for sintered Swarcoforce samples (Figure 11) in contrast to the previously investigated borosilicate glass [10]. As described in the previous sections, the green bodies and sintered parts possessed a pronounced brownish appearance. The sample with the lower thickness of 1.8 mm (Figure 11) showed a slight transmittance between 550 and 800 nm, while the thicker sample with 3.7 mm had almost no optical transmittance. This poor transmittance can be attributed to scattering and the general brownish appearance. For clarification, chemical analyses of the pristine Swarcoforce glass powder and the sintered glass powder were performed; the results can be seen in Table 6. There are only small deviations detectable. It is known from the literature that the presence of iron oxide and sodium sulfate can result in a brownish color in glass [27,28]. The chemical analyses showed that the glass used contained 6.89 wt% Na_2_O, 0.36 wt% SO_3_, and 0.65 wt% Fe_2_O_3_ in the initial glass filler. After sintering, the content of SO_3_ was reduced, while the content of Fe_2_O_3_ increased. The measured glass composition could offer certain evidence for the brownish coloring.

## 4. Conclusions

Within this work, new feedstock compositions consisting of soda-lime glass and partially water-soluble PEG/PVB binders were developed and successfully tested for replication in injection molding. Alongside the process chain of GIM, the most important results have been concluded as the following:

1. Glass containing feedstocks with a solid load of 55 Vol% and 60 Vol% were compounded and characterized by high pressure capillary rheology at 140 °C in a wide shear rate range. The impact of the average molecular mass of the PEGs on the flow behavior was estimated.
2. The principal feasibility of injection molding using the new feedstock systems targeting the realization of small and larger parts could be demonstrated successfully.
3. The essential parameters for the two-step debinding process were evaluated.
4. Optimized sintering parameters in air produced dense glass parts with no or minor internal defects, as characterized by nondestructive methods like CT.
5. Optical transmission spectroscopy showed that the samples possessed negligible transmittance properties.

## 5. Outlook

In powder injection molding with increasing part size, the replication process, especially when debinding and sintering, can be challenging with respect to obtaining dense and defect-free sintered parts. Therefore, one focus will be set on further parameter optimization along the whole process chain to avoid cracks and voids when considering parts of different sizes. In addition, a second focus should be the adaption of the described work with 3D printing, especially in Fused Filament Fabrication, and towards the realization of microfluidic devices, such as micro reaction technology.

## Figures and Tables

**Table 1:** The binder components used, their functions, and the related suppliers.

Binder Component	Function	Supplier
PEG4000	Plasticizer	Carl Roth GmbH + Co. KG, Karlsruhe, Germany
PEG8000	Plasticizer	Carl Roth GmbH + Co. KG, Karlsruhe, Germany
PEG20000	Plasticizer	Carl Roth GmbH + Co. KG, Karlsruhe, Germany
PVB B30H	Backbone Polymer	Kuraray Europe GmbH, Hattersheim a. M., Germany
SA	Surfactant/Release agent	Carl Roth GmbH + Co. KG, Karlsruhe, Germany

**Table 2:** Overview of investigated feedstock systems with different compositions.

Feedstock Denomination	Solid Load (Vol%)	PEG Type	PVB Type	PEG/PVB Ratio	SA Content (mg/m^2^)
SF1	55	4000	B30H	50:50	35
SF2	55	8000	B30H	50:50	35
SF3	55	20,000	B30H	50:50	35
SF4	60	4000	B30H	50:50	35
SF5	60	8000	B30H	50:50	35
SF6	60	20,000	B30H	50:50	35

**Table 3:** Overview of the equilibrium torque after 1 h at 125 °C, with a melt viscosity (140 °C) at two different shear rates typical for FFF (100 1/s) and injection molding (1000 1/s), as well as the viscosity ratio.

Feedstock	Equilibrium Torque (Nm)	Viscosity@100 1/s (Pa s)	Viscosity@1000 1/s (Pa s)	Viscosity Ratio
SF1	2.3	144	80	1.8
SF2	3.7	192	108	1.8
SF3	4.9	351	127	2.8
SF4	2.5	206	105	2.0
SF5	3.9	240	116	2.1
SF6	5.6	513	173	3.0

**Table 4:** Injection molding parameters for Swarcoforce-based feedstocks.

Feedstock	Melt Injection Temperature (°C)	Injection Speed (mm/s)	Cooling Time (s)
SF1–SF3	140	50	20
SF4–SF6	140	50	30

**Table 5:** Measured sinter densities of selected Swarcoforce feedstocks.

Feedstock	Solid Load (Vol%)	Sinter Density (% th.)	Considered Sample Numbers
SF2	55	95.6 ± 6.4	15
SF3	55	99.1 ± 1.6	12
SF6	60	100 ± 0.3	12

**Table 6:** Chemical analysis of Swarcoforce glass powder. All measured data were average values.

Material	Pristine (wt%)	Sintered (wt%)	Material	Pristine (wt%)	Sintered (wt%)
C	0.02	0.02	Cr_2_O_3_	0.05	0.09
Na_2_O	6.89	6.89	MnO	0.04	0.03
MgO	2.22	2.35	Fe_2_O_3_	0.65	0.79
Al_2_O_3_	0.89	1.10	ZnO	0.03	0.04
SiO_2_	70.2	70.8	SrO	0.05	0.05
SO_3_	0.36	0.09	ZrO_2_	0.02	0.16
K_2_O	0.64	0.62	MoO_3_	<0.02	0.04
CaO	17.5	16.6	PbO	0.06	0.05
TiO_2_	0.12	0.13	Sum:	99.75	99.85

## Data Availability

The original data presented in the study are openly available in KITOpen Repository https://doi.org/10.35097/cffncun0dddhf98m.

## Abbreviations

The following abbreviations are used in this manuscript:

BET	Brunauer–Emmett–Teller
CIM	Ceramic Injection Molding
CT	Computer Tomography
FFF	Fused Filament Fabrication
GIM	Glass Injection Molding
HDPE	High Density Polyethylene
IM	Injection Molding
LDPE	Low Density Polyethylene
MEX	Material Extrusion
MIM	Metal Injection Molding
PEG	Polyethylene Glycol
PIM	Powder Injection Molding
PMMA	Polymethylmethacrylate
PVB	Polyvinylbutyral
PW	Paraffin Wax
RPM	Revolutions Per Minute
SA	Stearic Acid
SF	Swarcoforce
Vol%	Volume%

## References

[1] Mauro J.C. (2014). Grand Challenges in Glass Science. Front. Mater..

[2] Quintero F., Penide J., Riveiro A., Del Val J., Comesaña R., Lusquiños F., Pou J. (2020). Continuous fiberizing by laser melting (Cofiblas): Production of highly flexible glass nanofibers with effectively unlimited length. Sci. Adv..

[3] Schmitz A., Kamin’ski J., Maria Scalet B., Soria A. (2011). Energy consumption and CO_2_ emissions of the European glass industry. Energy Policy.

[4] Butler J.H., Hooper P.D., Letcher T.M., Vallero D.A. (2019). Chapter 15—Glass Waste. Waste.

[5] https://www.schott.com/en-gb/expertise/glass-melting-and-hot-forming.

[6] Czepiel M., Bankosz M., Sobczak-Kupiec A. (2023). Advanced Injection Molding Methods: Review. Materials.

[7] German R.M. (1990). Powder Injection Molding.

[8] German R.M., Bose A. (1997). Injection Molding of Metals and Ceramics.

[9] Moritz T., Lenk R. (2009). Ceramic injection molding: A review of developments in production technology, materials and applications. Powder Inject. Mould. Int..

[10] Zuern M., Schrage A., Antusch S., Bohn N., Holzer P., Hanemann T. (2024). Development of a Polyethylene Glycol/Polymethyl Methacrylate-Based Binder System for a Borosilicate Glass Filler Suitable for Injection Molding. Materials.

[11] Mader M., Schlatter O., Heck B., Warmbold A., Dorn A., Zappe H., Risch P., Helmer D., Kotz F., Rapp B.E. (2021). High-throughput injection molding of transparent fused silica glass. Science.

[12] Hidalgo J., Conteras J.M., Berzal D., Jiménez-Morales A., Torralba J.M. Fabrication of Glass Components by Powder Injection Moulding (PIM) Recycling Glass Waster. Proceedings of the PM 2010 World Congress.

[13] Moritz T., Schilm J., Mannschatz A., Peschel M. (2014). Glass Powder Injection Molding—Ceramic Processing Applied to Glass Components. Ceram. Appl..

[14] German R.M., Hens K.F., Lin S.-T.P. (1991). Key Issues in Powder Injection Molding. Ceram. Bull..

[15] Bose A. (2025). Prof Randall M German: Shaping the field of Powder Injection Moulding—A MIM2025 tribute. Powder Inject. Molding Int..

[16] Hanemann T., Heldele R. (2011). Fatty Acid Surfactant Structure-Feedstock Flow Properties: Correlation for High-Pressure Ceramic Injection Molding. Int. J. Appl. Ceram. Technol..

[17] Thomas-Vielma P., Cervera A., Levenfeld B., Várez A. (2008). Production of alumina parts by powder injection molding with a binder system based on high density polyethylene. J. Europ. Ceram. Soc..

[18] Thavanayagam G., Pickering K.L., Swan J.E., Cao P. (2015). Analysis of rheological behaviour of titanium feedstocks formulated with a water-soluble binder system for powder injection moulding. Powder Technol..

[19] Hayat M.D., Goswami A., Matthews S., Li T., Yuan X., Cao P. (2017). Modification of PEG/PMMA binder by PVP for titanium metal injection moulding. Powder Technol..

[20] Hnatkova E., Hausnerova B., Hales A., Jiranek L., Derguti F., Todd I. (2017). Processing of MIM feedstocks based on Inconel718 powder and partially water-soluble binder varying in PEG molecular weight. Powder Technol..

[21] Eickhoff R., Antusch S., Nötzel D., Hanemann T. (2025). Metal fused filament fabrication (MF3) of Ti-6Al-4 V implants by using flexible, partially water-soluble binder systems. Mater. Des..

[22] Eickhoff R., Nötzel D., Oral G., Scholz M., Hanemann T. (2025). Ceramic fused filament fabrication (CF3) of zirconia implants by using flexible, partially water-soluble binder systems. Mater. Des..

[23] https://www.swarco-indusferica.com/swarcoforce.

[24] Weber O. (2015). Wasserlösliche Bindersysteme zum Minimieren von Pulver-Binder-Segregationseffekten im Mikropulverspritzguss. Doctoral Dissertation.

[25] Mezger T.G. (2014). The Rheology Handbook.

[26] https://www.goettfert.com/knowledge/applications/for-capillary-rheometer/weissenberg-rabinowitsch.

[27] https://www.glaeserundflaschen.de/steffi/dies-und-das/wissenswertes/wie-wird-buntes-glas-hergestellt.

[28] https://schaeferglas.de/blog/farbglas/.

